# Effects of Vasopressin Receptor Agonists during the Resuscitation of Hemorrhagic Shock: A Systematic Review and Meta-Analysis of Experimental and Clinical Studies

**DOI:** 10.3390/jpm13071143

**Published:** 2023-07-16

**Authors:** Eleni Laou, Nikolaos Papagiannakis, Androniki Papadopoulou, Theodora Choratta, Minas Sakellakis, Mariachiara Ippolito, Ioannis Pantazopoulos, Andrea Cortegiani, Athanasios Chalkias

**Affiliations:** 1 Department of Anesthesiology, Agia Sophia Children’s Hospital, 15773 Athens, Greece; elenilaou1@gmail.com; 2 First Department of Neurology, Eginition University Hospital, Medical School, National and Kapodistrian University of Athens, 11528 Athens, Greece; nikolas.papagia@gmail.com; 3 Department of Anesthesiology, G. Gennimatas General Hospital, 54635 Thessaloniki, Greece; andronikipapado@gmail.com; 4 Department of General Surgery, Metaxa Hospital, 18537 Piraeus, Greece; theodora.choratta@yahoo.com; 5 Department of Medical Oncology, Metropolitan Hospital, 18547 Piraeus, Greece; 6 Department of Surgical, Oncological and Oral Science (Di.Chir.On.S.), University of Palermo, 90133 Palermo, Italy; ippolito.mariachiara@gmail.com (M.I.); cortegiania@gmail.com (A.C.); 7 Department of Emergency Medicine, Faculty of Medicine, University of Thessaly, 41500 Larisa, Greece; pantazopoulosioannis@yahoo.com; 8 Department of Anesthesiology, Faculty of Medicine, University of Thessaly, 41500 Larisa, Greece; 9 Institute for Translational Medicine and Therapeutics, University of Pennsylvania Perelman School of Medicine, Philadelphia, PA 19104-5158, USA; 10 Outcomes Research Consortium, Cleveland, OH 44195, USA

**Keywords:** vasopressin, vasopressin receptor agonist, hemorrhagic shock, resuscitation, hemodynamics, outcome

## Abstract

Background: The clinical impact of vasopressin in hemorrhagic shock remains largely unknown. Objective: This systematic review and meta-analysis was designed to investigate the effects of vasopressin receptor agonists during the resuscitation of hemorrhagic shock. Methods: A systematic search of PubMed (MEDLINE), Scopus, and PubMed Central was conducted for relevant articles. Experimental (animal) and clinical studies were included. The primary objective was to investigate the correlation of vasopressin receptor agonist use with mortality and various hemodynamic parameters. Results: Data extraction was possible in thirteen animal studies and two clinical studies. Differences in risk of mortality between patients who received a vasopressin receptor agonist were not statistically significant when compared to those who were not treated with such agents [RR (95% CI): 1.17 (0.67, 2.08); *p* = 0.562; I^2^ = 50%]. The available data were insufficient to conduct a meta-analysis assessing the effect of vasopressin receptor agonists on hemodynamics. Drawing safe conclusions from animal studies was challenging, due to significant heterogeneity in terms of species and dosage of vasopressin receptor agonists among studies. Conclusions: Differences in risk of mortality between patients who received a vasopressin receptor agonist were not statistically significant when compared to those who were not treated with such agents after hemorrhagic shock. More data are needed to deduce certain conclusions.

## 1. Introduction

Massive trauma remains the leading cause of mortality among individuals under 45 years of age, with approximately 30–50% of deaths attributed to hemorrhagic shock [1,2]. The early phase of hemorrhage is characterized by a vasoconstrictive response, but if left untreated, it can progress to cardiovascular failure and vasodilation that is not responsive to conventional resuscitation strategies. During the progression of the condition, patients experience a life-threatening acute reduction in oxygen delivery to tissues, reaching levels below what is needed to maintain cellular homeostasis [3].

The resuscitation of hemorrhagic shock typically includes crystalloid infusion, administration of blood/blood products, and the use of vasopressors [4]. However, the role of the latter remains controversial, and many authors do not recommend their use [5,6], while others advocate for the early administration of vasopressors to minimize total fluid volume [7,8]. To date, no definitive recommendations exist regarding the optimal timing, type, and dosage of vasopressors in patients with hemorrhagic shock.

Vasopressin was first discovered in 1895 and was initially used in the treatment of diabetes insipidus. Since then, it has been extensively studied as an adjunctive therapy in the management of non-hemorrhagic circulatory failure. Of note, the fluctuation of vasopressin levels in response to shock is highly intriguing. Initially, a 10-fold increase in endogenous vasopressin levels is observed, which returns to baseline after a short period of time [9,10]. In addition, vasopressin is a key regulatory hormone participating in several homeostatic functions, including osmoregulation and cardiovascular control.

Trauma-induced hypovolemic shock remains difficult to treat and vasopressin has emerged as a potential pharmacologic adjunct [9,11]. Nevertheless, the precise impact on these patients remains unknown. The objective of this systematic review and meta-analysis was to investigate the effects of vasopressin receptor agonists during the resuscitation of hemorrhagic shock.

## 2. Materials and Methods

### 2.1. Protocol and Registration

The protocol was registered in the PROSPERO international prospective register of systematic reviews on 9 May 2023 (CRD42023422429). This systematic review and meta-analysis was reported according to the Preferred Reporting Items For Systematic Reviews And Meta-Analyses (PRISMA) checklist (Table S1) [12].

### 2.2. Inclusion and Exclusion Criteria

Experimental (animal) and clinical (randomized and non-randomized controlled trials, comparative, cohort, validation, observational) studies investigating the effects of vasopressin receptor agonists during the resuscitation of hemorrhagic shock were included. Exclusion criteria were review articles, case studies, and non-English literature. The comparators were either the administration of fluids without vasopressin receptor agonists or the administration of norepinephrine.

### 2.3. Outcomes of Interest

#### 2.3.1. Primary Outcomes

##### Experimental Studies

The primary outcome was to investigate (1) mortality within 24 h after resuscitation and (2) the relationship of vasopressin receptor agonists with hemodynamic parameters [heart rate, mean arterial pressure (MAP), central venous pressure (CVP), cardiac output (CO) or cardiac index (CI), systemic vascular resistance (SVR) or systemic vascular resistance index (SVRI)] during the resuscitation of hemorrhagic shock.

##### Clinical Studies

The primary outcome was to investigate (1) mortality at hospital discharge, at 30 days, at 90 days, and at 1 year and (2) the relationship of vasopressin receptor agonists with hemodynamic parameters [heart rate, mean arterial pressure (MAP), central venous pressure (CVP), cardiac output (CO) or cardiac index (CI), systemic vascular resistance (SVR) or systemic vascular resistance index (SVRI)] during the resuscitation of hemorrhagic shock.

#### 2.3.2. Secondary Outcomes

##### Experimental Studies

Association of vasopressin receptor agonists with amount of administered fluids, coagulopathy, end-organ damage, multiple organ failure.

##### Clinical Studies

Association of vasopressin receptor agonists with amount of administered fluids, coagulopathy, end-organ damage, multiple organ failure, days of mechanical ventilation, intensive care unit (ICU) length of stay, and hospital length of stay.

### 2.4. Search Strategy

The search strategy intended to explore all available published experimental (animal) and clinical studies from inception up to 30 April 2023, and was designed by four authors (EL, MI, ACo, ACha). A comprehensive initial search was employed in PubMed (MEDLINE), Scopus, and PubMed Central for articles containing any of the following terms in the abstract or title: the MeSH^®^ terms hemorrhagic shock, hemorrhage, hypovolemic shock, vasopressin, vasopressin analogue; the wildcard terms hemorr*, vasopr*; or any of the following terms: arginine vasopressin, argipressin, AVP, vasopressin receptor, V_1_ agonist, V_2_ agonist, terlipressin, lysine vasopressin, lypressin, phenypressin. We then searched for those articles which met the above criteria and contained any of the following MeSH^®^ terms: cardiovascular dynamics, hemodynamics, preload, venous return, heart-lung interactions, cardiac output, cardiac index, stroke volume, systemic vascular resistance, afterload, arterial pressure, pulse pressure, blood flow, perfusion, or microcirculation; or the following non-MeSH terms: complications, outcome, mortality, or survival (Table S2). Another search was conducted with the reference lists of all identified reports and articles for additional studies, and a grey literature search was conducted on Google Scholar. The ClinicalTrials.gov website was also searched for all articles containing any of the following terms: vasopressin, arginine vasopressin, argipressin, vasopressin receptor, V_1_ agonist, V_2_ agonist, terlipressin, lysine vasopressin, lypressin, phenypressin, hemorrhage, and hemorrhagic shock. The last literature search was performed on 10 June 2023.

### 2.5. Data Extraction

The titles and abstracts of studies obtained using the search strategy and those from additional sources were independently screened by four review authors (EL, AP, TC, MS) to identify studies that potentially meet the inclusion criteria outlined above. The data from each study were independently extracted by three review authors (EL, NP, IP) with a customized format. Disagreements regarding study eligibility were resolved through discussions among the authors.

A standardized proforma was used to extract data from the included studies, enabling the assessment of study quality and evidence synthesis. Extracted information included: publication details (authors, year), study information (design, population), all-cause mortality in all groups at hospital discharge, 30 days, 90 days, and 1 year, presence of multiple organ failure, hemodynamic profile (heart rate, mean arterial pressure, central venous pressure, lactate levels, urinary output, cardiac output, oxygen delivery/consumption), adverse events, vasopressor-free days, duration of mechanical ventilation, ICU length of stay, hospital length of stay, and information for assessment of the risk of bias. Discrepancies were resolved through discussion or with the input of the other authors if necessary. The authors of studies with missing data were contacted in an attempt to obtain the relevant data.

### 2.6. Assessment of Methodological Quality

Articles identified for retrieval were assessed by three independent authors (EL, NP, IP) for methodological quality before inclusion in the review using a standardized critical appraisal tool. Any disagreements between the authors during the process of appraising the articles were resolved through discussion involving all the authors. The quality of the included observational studies was assessed using the MINORS tool, while the Risk of Bias 2.0 (RoB 2.0) tool was used for RCTs.

### 2.7. Data Analysis and Synthesis

A meta-analysis was used to estimate the pooled risk ratios (RR), along with their 95% Confidence Interval (95% CI), if two or more studies had complete data about mortality in both groups. The meta-analysis was conducted using random effect models. The statistical heterogeneity was estimated by using the Cochran’s Q and I^2^ indices. The statistical significance was set at *p* < 0.05. All statistical analyses were performed in R v4.0.

Subgroup analysis was planned and would be conducted (data permitting) according to the type of vasopressin receptor agonists, degree of blood loss according to the American College of Surgeons Advanced Trauma Life Support (ATLS) hemorrhagic shock classification, and cardiorespiratory comorbidities (coronary artery disease, heart failure, pulmonary arterial hypertension, chronic lung disease).

## 3. Results

**Figure 1:**
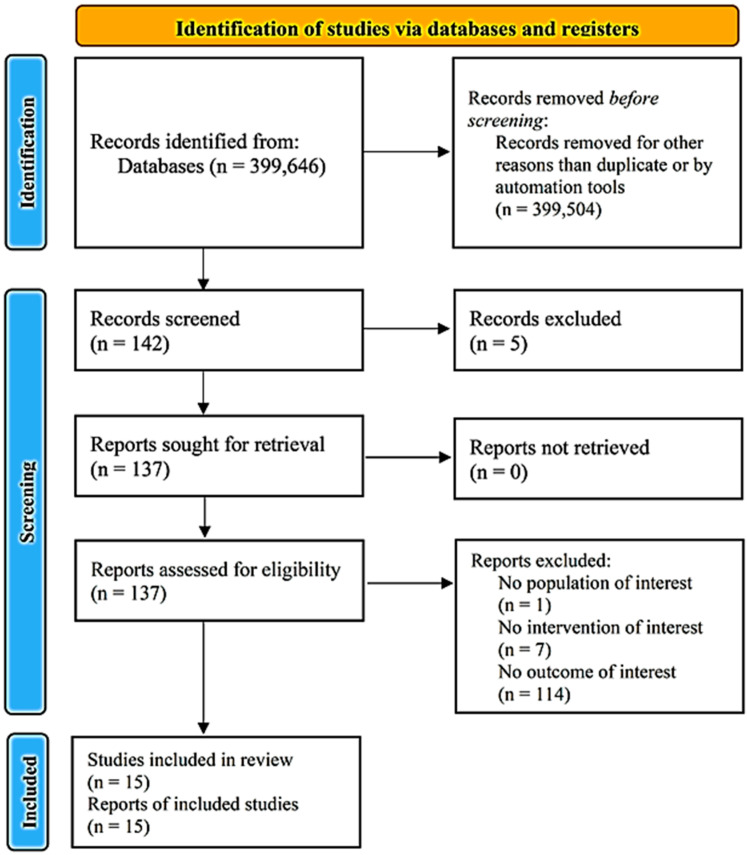
Preferred Reporting Items for Systematic Reviews and Meta-Analyses (PRISMA) diagram.

Altogether, 137 relevant citations were identified and screened. Of the 137 citations, 50 experimental and 6 clinical studies were selected for full review based on their abstract and were included in our final assessment for possible data extraction (Figure 1). In total, data extraction was feasible in 13 animal studies (Table 1) [11,13,14,15,16,17,18,19,20,21,22,23,24] and 2 clinical studies (Table 2) [25,26].

### 3.1. Risk of Bias, Quality of Evidence

**Figure 2:**
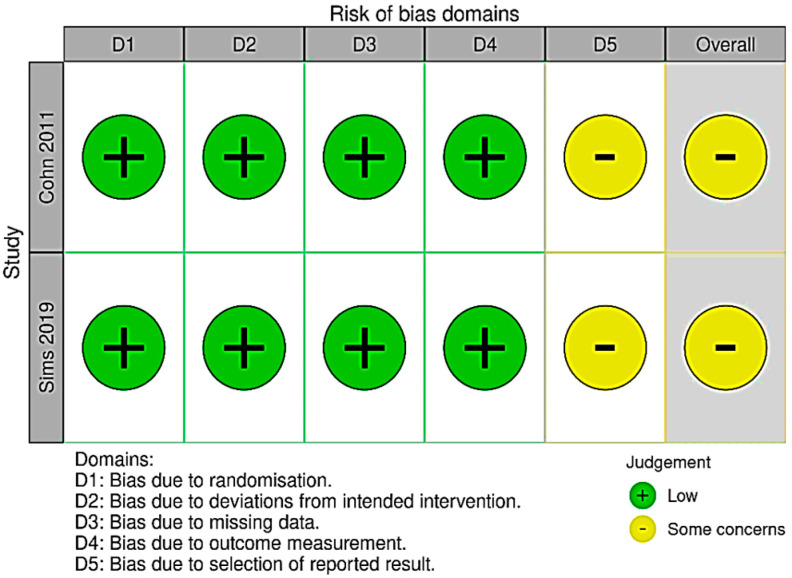
Traffic lights plot for risk of bias assessment.

The quality of the included observational studies was assessed using the MINORS tool, while the Risk of Bias 2.0 (RoB 2.0) tool was used for RCTs (Figure 2). Data were assessed for heterogeneity using the I^2^ statistic interpreted using the guidance from the Cochrane Handbook for Systematic Reviews of Interventions. Assessment of potential publication bias was carried out by visual inspection of funnel plots. *p* values < 0.05 were considered statistically significant.

### 3.2. Synthesis including All Data

Two studies, a double-blind, randomized, parallel-group, controlled trial and a randomized, double-blind placebo-controlled trial, were identified, with included data on 30-day mortality in patients who received vasopressin receptor agonists compared to those who did not, resulting in a total population of 179 patients (Table 2) [25,26]. In both studies, patients received a 4 IU-bolus of vasopressin receptor agonist followed by an infusion of ~0.04 IU min^−1^ for 5–48 h.

**Figure 3:**
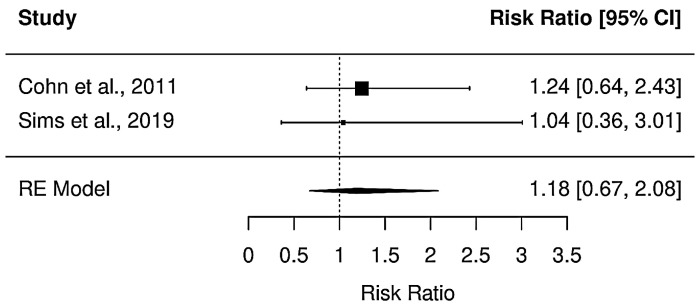
Effect of vasopressin receptor agonist on patients with hemorrhagic shock. Presented in plots Standardized Mean Difference and 95% confidence intervals. Positive values (less than zero) favor intervention. Data are from references [25,26].

The differences in the risk of mortality between patients who received a vasopressin receptor agonist were not statistically significant when compared to those who were not treated with such agents after hemorrhagic shock [RR (95% CI): 1.17 (0.67, 2.08); *p* = 0.562]. Medium heterogeneity was present (I^2^ = 50%). Forest plots for the different measurements are presented in Figure 3 and a summary of the results is presented in Table 3.

#### Relationship of Vasopressin Receptor Agonists with Hemodynamic Parameters

The data were insufficient for conducting a meta-analysis regarding the effects of vasopressin receptor agonists on hemodynamics.

##### Characteristics of Experimental (Animal) Studies

The effect of vasopressin receptor agonists on cardiovascular dynamics in animal studies is depicted in Table 1. Safe conclusions could not be derived due to significant heterogeneity in terms of species and the dosage of vasopressin receptor agonist administration among studies. In general, eight studies showed that the administration of vasopressin receptor agonists may be effective in hemorrhagic shock [11,14,15,16,19,20,23,24], one study reported that arginine vasopressin (AVP) was comparable to norepinephrine with respect to hemodynamics and blood gases [17], and one study reported that vasopressin receptor agonists may have a detrimental effect on hemodynamic and metabolic function [22].

##### Characteristics of Clinical Studies

In one study, mean systolic blood pressure was numerically higher in the experimental group following the 5-h vasopressin infusion compared to the control group, but the mean difference between treatments was not statistically significant (*p* = 0.54) [25]. In the other study, AVP maintained a higher MAP but differences between groups were not statistically significant [26]. Similarly, although the AVP group received a lower volume of vasopressors, these differences did not reach statistical significance [26].

### 3.3. Secondary Outcomes

In general, the data were insufficient for assessing all the secondary outcomes.

#### 3.3.1. Experimental (Animal) Studies

We identified seven studies investigating the effects of AVP in different species. Among them, two studies (in hamsters and dogs) showed that AVP may improve oxygenation and metabolic variables [20,22], one (conducted in swine) reported comparable brain metabolism in both groups (VAP vs. norepinephrine) [17], one study (in swine) indicated that AVP may decrease pH and increase lactate (tissue hypoperfusion) [18], and three studies (one in swine, two in rats) reported that the differences between groups were not statistically significant (Table 4) [11,13,21].

#### 3.3.2. Clinical Studies

The administration of vasopressin receptor agonists may decrease the total volume of blood/blood products and fluids required after hemorrhagic shock (Table 5). In the study by Cohn et al., three (30.0%) patients in the control and two (40%) in the vasopressin arm suffered cardiopulmonary failure/arrest, while no significant differences were observed with respect to ventilator-free days (*p* = 0.976) or multiple organ dysfunction syndrome (*p* = 0.499) [25].

In the study by Sims et al., AVP did not significantly affect the overall complication rate (*p* = 0.44) or resuscitation-related complications, such as acute respiratory distress syndrome (*p* = 031) and acute kidney injury (*p* = 0.19). Although patients in the AVP group had a lower positive fluid balance at 48 h (*p* = 0.03), this did not significantly alter the incidence of the overall or resuscitation-related complication rate (*p* = 0.37). However, it was associated with a decreased incidence of deep venous thrombosis (*p* = 0.02). The duration of mechanical ventilation (*p* = 0.43), ICU length of stay (*p* = 0.06), and hospital length of stay (*p* = 0.12) were not affected by AVP [26].

## 4. Discussion

This systematic review and meta-analysis, encompassing experimental and clinical studies, found that differences in the risk of mortality between patients who received a vasopressin receptor agonist were not statistically significant when compared to those who were not treated with such agents after hemorrhagic shock. The data were insufficient for conducting a meta-analysis regarding the effects of vasopressin receptor agonists on hemodynamics in the clinical setting. Safe conclusions could not be derived from animal studies due to the significant heterogeneity in species and the dosage of the vasopressin receptor agonist.

Vasopressor use in hemorrhagic shock represents an area that is in critical need of high-quality evidence. Norepinephrine is the most commonly used medication, but its use may be limited in patients with increased endogenous catecholamine levels or in the presence of acidic conditions [4,27,28]. Indeed, several studies described an increase in mortality or no significant benefit when this agent is used in cases of traumatic hemorrhage [27,29,30,31,32]. Thus, several experts have expressed concerns regarding the appropriateness of its use as well as the potential selection of alternative agents [33].

Vasopressin is a hormone secreted by the posterior pituitary gland in response to increased serum osmolality or hypotension [34,35]. However, it has a half-life of only 4–35 min and may become depleted relatively soon after the onset of bleeding [21,36], leading to catecholamine resistance, increased venous capacitance, and shock [37]. The relatively short half-life is probably the most likely reason that most studies used a bolus dose followed by infusion or solely an infusion, in order to maintain its activity.

Theoretically, exogenous low-dose vasopressin may be effective in improving vascular tone and perfusion pressure, especially in patients with low endogenous levels [38,39]. Thus, the use of this agent as an adjunct treatment in patients with hemorrhagic shock is intriguing. A previous systematic review and meta-analysis of randomized animal trials published in 2014 reported that AVP and terlipressin improve survival in the early phases of hemorrhagic shock compared to other vasopressor drugs [40]. However, the included experimental studies measured survival rates at different time points, while the dosages were higher than those used in human studies. In addition, these results have not been confirmed by clinical trials. On the contrary, the present systematic review and meta-analysis found that differences in risk of mortality between patients who received a vasopressin receptor agonist were not statistically significant when compared to those who were not treated with such agents after hemorrhagic shock. Despite the degree of heterogeneity in our study, this finding raises concerns regarding the optimal use of vasopressin receptor agonists in patients with traumatic hemorrhage.

Previous research has shown that early vasopressin use in patients with hemorrhagic shock may decrease the volume of required total blood/blood products [25,40], and the study by Sims et al. confirmed this notion [total blood products 1.4 (0.5–2.6) vs. 2.9 (1.1–4.8) L, *p* = 0.01] [26]. Sims et al. also reported that both the AVP and placebo group received relatively large amounts of crystalloid fluids within 48 h [9.9 (7.9–13) vs. 11 (8.9–15) L; difference, −1.07 (−3.04–0.62), *p* = 0.22], but the ratio of fluid total input to total output was higher in the latter [5.0 (2.5–7.0) vs. 6.7 (4.0–11.4), *p* = 0.03] [26]. In that study, AVP maintained a higher MAP, but the differences in hemodynamics and 48-h total volume of vasopressors [0.4 (0.0 to 5.9) vs. 1.4 (0.2 to 7.6) g; difference, −0.23 (−1.37 to 0.53), *p* = 0.22] between groups were not statistically significant [26]. In addition, Cohn et al. reported that mean systolic blood pressure was higher in the vasopressin group than in the control group following the 5-h drug infusion, but the mean difference between treatments was not statistically significant [25]. We found only one animal study reporting data on hemodynamics and mortality. Dickson et al. used a swine polytrauma model with hemorrhagic shock to evaluate the effects of vasopressin (0.4 IU kg^−1^ per bolus) on blood pressure and cerebral blood flow during initial fluid resuscitation [24]. In this experimental model, increasing blood pressure with vasopressin [44.1 (17.4) vs. 28.6 (10.6)] did not improve brain or vital organ perfusion, likely due to increased internal blood loss, and did not affect survival at 6 h [145.8 (94) min vs. 137.4 (39.7) min]. Unfortunately, safe conclusions cannot be drawn from the included animal studies due to the significant heterogeneity with respect to species and the dosage of vasopressin receptor agonist among studies. Furthermore, the clinical data are also not sufficient to deduce safe conclusions.

Whether patients who are treated with hypotensive resuscitation (permissive hypotension) can benefit from vasopressin administration remains unknown given the different methods of achieving it and the associated challenges. In these individuals, volume repletion is limited in order to sustain a goal systolic arterial pressure or MAP below normal physiological conditions during hemorrhage. Indeed, several studies indicate a decrease in mortality when blood pressure is adjusted below the normal physiologic range [4], although it should be kept within a specific range to maintain microvascular autoregulation, i.e., the intrinsic capacity of resistance vessels in end-organs to dilate and constrict in response to dynamic perfusion pressure changes, especially in patients with comorbidities. Considering that hypotensive resuscitation is mainly used in the early stages of hemorrhage to rapidly stabilize the patients and is not recommended after bleeding is controlled, it is unlikely that these individuals have low endogenous vasopressin levels and benefit from exogenous administration. However, the latter could be considered carefully, and on a case-by-case basis, e.g., when vasopressors are needed to maintain the arterial pressure and perfusion targets in acidotic patients or those with pulmonary hypertension who are treated with permissive hypotension.

In the present analysis, we identified two animal studies showing that AVP may possibly improve oxygenation and metabolic variables [20,22] and five reporting negligible or detrimental effects on hemodynamic and metabolic function [11,13,17,18,21]. However, there are significant translational concerns raised by the fact that AVP does not significantly affect the overall complication rate, organ injury, duration of mechanical ventilation, multiple organ dysfunction syndrome, ICU length of stay, and hospital length of stay in the clinical setting [25,26].

## 5. Limitations

This analysis investigated the effects of vasopressin receptor agonists in the resuscitation of hemorrhagic shock. First, the data were insufficient for assessing all the pre-specified outcomes or performing subgroup analysis. Furthermore, it was likely that the included animal studies encompass a highly heterogeneous group in terms of species and dosages. Due to heterogeneity and lack of data, the synthesis of all the available knowledge on specific outcomes was difficult for animal studies. Another issue was the lack of multiple randomized controlled trials; therefore, the synthesis of all the available knowledge on the specific outcomes was based on two studies only. Heterogeneity may have also limited the results of the meta-analysis of clinical studies, as our intervention consisted of different vasopressin receptor agonists. Consequently, the conclusions drawn from this review and the clinical implications of the findings must be interpreted with caution.

## 6. Conclusions

This systematic review and meta-analysis found that the risk of mortality between patients who received a vasopressin receptor agonist and those who did not was not statistically significant. The available data are not enough to deduce certain conclusions.

## 7. Perspectives

The optimal timing of vasopressor administration in patients with hemorrhagic shock is still heavily debated and often relies on subjective clinical assessments. Some experts believe that the early addition of a vasopressor results in higher complication rates or mortality while others favor their early use to minimize the total amount of administered fluids. However, cardiovascular reserves vary with age and/or comorbidities and an individualized, physiology-guided approach is necessary to maintain a balance between circulatory volume and vascular tone and restore perfusion and tissue oxygenation [1].

A rapid decrease in circulating blood volume decreases stressed volume and promotes volume recruitment from the splanchnic circulation. Hemodynamic parameters may remain relatively stable during the initial stages of hemorrhage (10–12%), but a decrease in arterial pressure and/or cardiac output subsequently ensue. Clinically noticeable hypovolemia occurs when stressed volume starts to decrease, while the unstressed volume has decreased to a physiologically possible maximum. In the latter case (severe hypovolemia), veins are usually maximally constricted and exogenous pure alpha-1 adrenergic agonists (e.g., phenylephrine) would not further increase venoconstriction and will only constrict arteries [41,42]. Of note, the activation of the abundant alpha-adrenergic receptors in the (already constricted) hepatic veins increases the impedance of the outflow of blood from the splanchnic system into systemic circulation, which leads to the sequestration of blood within the liver [43,44,45,46,47], possibly depriving a valuable amount of volume (autotransfusion) from the systemic circulation (venous return), while further constriction of arteries aggravates tissue perfusion and hypoxia. Compared to alpha-1 adrenergic agonists, norepinephrine might exert an additional benefit by stimulation of beta-2 adrenoceptors, facilitating the emptying of the splanchnic venous system, but it may also decrease pulmonary vascular compliance [45,46]. In addition, catecholamines are associated with harmful effects, such as metabolic stress and immunomodulation, and alternative therapeutic strategies and concepts, e.g., “decatecholaminization”, are needed [48].

Vasopressin has little effect on the venous system [49,50], but it may indirectly affect both the venous return and cardiac output. Preclinical evidence suggests that it can significantly reduce mesenteric flow without changing cardiac output [51], but it may also shift blood downstream towards the heart with a resultant increase in venous return and cardiac output. In general, it is difficult to predict which of the two actions will prevail. Although several experimental studies favor the use of vasopressin in massively hemorrhaging patients compared with other pharmacologic alternatives [52,53,54], our study cannot confirm these findings in the clinical setting due to the scarcity of the available data.

The key point in patients with hemorrhagic shock who do not respond to exogenous catecholamines may be the endogenous vasopressin levels. Vasopressin appears to exert its osmoregulatory effects within the normal circulating levels of 1 to 7 pg mL^−1^, while at much higher levels (100 to 300 pg mL^−1^), it begins to have a vasopressor effect [9]. Administration of vasopressin may be ineffective in individuals with normal or increased endogenous levels, leading to significant adverse events, e.g., end-organ ischemia. In these patients, the activation of V_2_ receptors by endogenous vasopressin promotes water reabsorption, and therefore fluid resuscitation should be carefully titrated, particularly in prior fluid-intolerant individuals, such as those with preexisting heart failure or chronic kidney disease, because they may be unable to tolerate even small to moderate amounts of fluids [55,56].

With prolonged hypotension, however, endogenous vasopressin levels are depleted, leading to vasodilation and tissue hypoperfusion. In these patients, who are usually acidotic, exogenous vasopressin receptor agonists may be beneficial and improve end-organ perfusion. Also, low-dose vasopressin may stimulate corticotropin secretion and enhance diuresis and renal function, improving hemodynamics during resuscitation of hemorrhagic shock [56,57]. At the microcirculatory and molecular levels, treatment with low-dose vasopressin in combination with fluid therapy may improve organ perfusion and mitochondrial function [20,58].

Further research is urgently needed to determine and fully exploit the potential benefits of vasopressin use in hemorrhagic shock. The available experimental evidence mandates the necessity to think and get used to acting “outside the box”. For example, as patients with vasopressin deficiency have their vasopressin receptors unoccupied [9], co-administration of vasopressin receptor agonists with a vasodilator (e.g., nitroprusside) may improve both macrohemodynamics and microcirculatory blood flow [22,59]. Specific recommendations are needed to bring a new level of standardization to animal models of hemorrhagic shock and ultimately to improve the translation of experimental findings. We strongly believe that translational research should be advanced to a highly relevant, standardized, and validated experimental critically ill swine model.

## Figures and Tables

**Table 1:** Effect of vasopressin receptor agonist on cardiovascular dynamics.

Author Name, Year	Species	VRA/ Comparator	Dosage	Variable				
				CO/CI	SVR/SVRI	SV	CVP/RAP	MAP
Stadlbauer et al., 2003 [13]	Swine	AVP/Ringer’s and 3% gelatine solution	0.4 IU kg^−1^ + infusion 0.08 IU kg^−1^ min^−1^	NA	NA	NA	NA	72 (26) vs. 38 (16)
Jochem et al., 2004 [14]	Rat	AVP/Saline	0.25 nmol kg^−1^	11.5 (2.19) vs. 5 (0.44)	5.01 (0.22) vs. 4.02 (0.25) *	NA	NA	57.7 (6.1) vs. 20.1 (2.8)
Yoo et al., 2006 [15]	Dog	Vasopressin/ Saline	0.4 IU kg^−1^	5.33 (0.44) vs. 6.67 (0.86)	1485 (174) vs. 1502 (228)	NA	NA	NA
Johnson et al., 2006 [16]	Rat	AVP/Ringer’s	0.05 IU kg^−1^ min^−1^	NA	2386 (295) vs. 1362 (316)	NA	4 (1) vs. 7 (2)	NA
Meybohm et al., 2007 [17]	Swine	AVP/Norepinephrine	0.4 IU kg^−1^	NA	NA	NA	NA	49 (16) vs. 39 (19)
Stadlbauer et al., 2007 [18]	Swine	AVP/Ringer’s and 3% gelatine solution	0.4 IU kg^−1^ + infusion 0.08 IU kg^−1^ min^−1^	NA	NA	NA	NA	60 vs. 55 ^#^
Li et al., 2011 [19]	Rat	AVP/Ringer’s	0.4 IU kg^−1^	NA	NA	NA	NA	39.8 (2) vs. 41.3 (1.1)
Lima et al., 2012 [20]	Hamster	AVP/Saline	0.4 IU kg^−1^	NA	NA	NA	NA	73.56 (12.51) vs. 72.5 (15.07)
Liu et al., 2013 [11]	Rat	AVP/Ringer’s	NA	NA	NA	NA	NA	36.25 (3.34) vs. 37.94 (2.74)
Sims et al., 2017 [21]	Rat	AVP/Ringer’s	0.5 IU kg^−1^ + 0.03 IU kg^−1^ min^−1^	NA	NA	NA	NA	125 vs. 70
Dickson et al. 2018 [24]	Swine	AVP/No fluids	0.4 IU kg^−1^ + 0.4 IU kg^−1^ after 40 min	NA	NA	NA	NA	44.1 (17.4) vs. 28.6 (10.6)
Truse et al., 2019 [22]	Dog	AVP/Saline	0.001 ng kg^−1^ min^−1^	↓ CO ^#^	NA	23 (3) vs. 23 (2)	NA	NA
Gil-Anton et al., 2020 [23]	Swine	Terlipressin/ Saline	20 mg kg^−1^	3.4 (0.5) vs. 3.2 (0.4)	1361 (227) vs. 1157 (217)	NA	6 (1) vs. 6 (1)	65 (8) vs. 53 (5)

VRA, vasopressin receptor agonist; AVP, arginine vasopressin; CO, cardiac output; CI, cardiac index; SVR, systemic vascular resistance; SVRI, systemic vascular resistance index; SV, stroke volume; CVP, central venous pressure; RAP, right atrial pressure; MAP, mean arterial pressure. * Total peripheral resistance index (TPRI). # MAP declined more rapidly in the fluid resuscitation group than in AVP-treated swine after 5 min of experimental therapy. # Dose escalation of AVP led to an increase in afterload with a dose-dependent rise in SVR and MAP and a reduced heart rate at constant SV. This was associated with a decrease in CO from 84 ± 8 mL kg^−1^ min^−1^ to 78 ± 6 mL kg^−1^ min^−1^ (0.1 ng kg^−1^ min^−1^ AVP) and 68 ± 6 mL kg^−1^ min^−1^ (1 ng kg^−1^ min^−1^ AVP), respectively.

**Table 2:** Effect of vasopressin receptor agonist on mortality and adverse events following hemorrhagic shock.

Author, Year	Type of Study	Type of Injury	VAR/Comparator	Dosage	End-Organ Damage/MOF	Mortality at 30 Days
Cohn et al., 2011 [25]	Double-blind, randomized, parallel-group, controlled trial	Acute traumatic injury	Vasopressin/Saline	4 IU bolus + 2.4 IU h ^−1^ for 5 h	No significant differences	13/38 vs. 11/40, *p* = 0.52
Sims et al., 2019 [26]	Randomized, double-blind placebo-controlled clinical trial	Acute traumatic injury	AVP/Saline placebo	4 IU bolus or placebo + ≤0.04 IU min^−1^ or placebo for 48 h	No significant differences	6/49 vs. 6/51, *p* = 0.94

VAR, Vasopressin receptor agonist; AVP, arginine vasopressin.

**Table 3:** Summary of meta-analysis results.

Parameter	Number of Studies	N (Total)	Estimate (SMD)	*p*-Value	95% CI	I^2^	Q	*p* (Q)
Mortality at 30 days	2	179	0.17	0.562	0.67 to 2.08	50%	1.06	0.586

**Table 4:** Effect of vasopressin receptor agonist on oxygenation and metabolic variables in animal studies.

Author, Year	Species	VRA/ Comparator	Dosage	Variable						
				pH	PaO_2_	PaCO_2_	HCO_3_	BD	Lactate	SaO_2_
Stadlbauer et al., 2003 [13]	Swine	AVP/Ringer’s and 3% gelatine solution	0.4 IU kg^−1^ + infusion 0.08 IU kg^−1^ min^−1^	7.44 (0.11) vs. 7.27 (0.05)	199 (161) vs. 179 (37)	26 (7) vs. 36 (8)	NA	NA	9.5 (3.1) vs. 9.0 (0.7)	NA
Meybohm et al., 2007 [17]	Swine	AVP/Norepinephrine	10 IU bolus + 2 IU kg^−1^ h^−1^	7.24 (0.06) vs. 7.25 (0.05)	NA	NA	NA	−7.5 (3.5) vs. −5.9 (4.9)	NA	NA
Stadlbauer et al., 2007 [18]	Swine	AVP/Ringer’s and 3% gelatine solution	0.4 IU kg^−1^ + infusion 0.08 IU kg^−1^ min^−1^	7.15 (0.05) vs. 7.51 (0.01)	312 (134) vs. 239 (131)	30 (3) vs. 24 (7)	NA	−8.8 (5.8) vs. −8.9 (3.3)	11.1 (3.11) vs. 8.44 (2.66)	NA
Lima et al., 2012 [20]	Hamster	AVP/Saline	0.0001 IU kg^−1^ min^−1^	7.40 (0.05) vs. 7.38 (0.11)	84.3 (19.41) vs. 113.4 (28.6)	42.31 (7.85) vs. 41.2 (8.81)	26.1 (3.8) vs. 24.7 (4.8)	1.20 (3.99) vs. −0.30 (5.98)	1.85 (1.04) vs. 3.75 (3.08)	NA
Liu et al., 2013 [11]	Rat	AVP/Ringer’s	0.4 U kg^−1^	7.39 (0.032) vs. 7.39 (0.05)	118.4 (9.7) vs. 121.1 (10.1)	32 (2.9) vs. 34.1 (1.9)	NA	−3.81 (2.81) vs. −4.43 (2.08)	NA	NA
Sims et al., 2017 [21]	Rat	AVP/Ringer’s	0.5 IU kg^−1^ + 0.03 IU kg^−1^ min^−1^	7.37 (0.05) vs. 7.35 (0.10)	107 (11) vs. 118 (35)	34 (4) vs. 31 (9)	NA	NA	25 (5) vs. 25 (5)	NA
Truse et al., 2019 [22]	Dog	AVP/Saline	0.001–1 ng kg^−1^ min^−1^	7.39 (0.01) vs. 7.38 (0.02)	NA	36 (1) vs. 36 (2)	21 (0.6) vs. 20.2 (0.6)	NA	1.2 (0.3) vs. 1.9 (0.6)	99 (0.2) vs. 98 (0.2)

VRA, vasopressin receptor agonist; AVP, arginine vasopressin.

**Table 5:** Effect of vasopressin receptor agonist on transfusion and fluid administration.

Author, Year	Type of Study	Type of Injury	VAR/Comparator	Dosage	Fluid	
					Type	Dose
Cohn et al., 2011 [25]	Double-blind, randomized, parallel-group, controlled trial	Acute traumatic injury	Vasopressin/Saline	4 IU bolus + 2.4 IU h ^−1^ for 5 h	Crystalloids	13.2 ± 9.8 L vs. 16 ± 12.8 L *
					Blood and blood products	3.8 ± 5 L vs. 5.4 ± 6.6 L
Sims et al., 2019 [26]	Randomized, double-blind placebo-controlledclinical trial	Acute traumatic injury	AVP/Saline placebo	4 IU bolus or placebo + ≤0.04 IU min^−1^ or placebo for 48 h	Crystalloids	5.0 [IQR, 2.5–7.0] vs. 6.7 [IQR, 4.0–11.4] L, *p* = 0.03
					Blood and blood products	1.4 [IQR, 0.5–2.6] vs. 2.9 [IQR, 1.1–4.8] L; *p* = 0.01

* Over the first 120 h (5 days). VAR, vasopressin receptor agonist; AVP, arginine vasopressin.

## Data Availability

Data can be made available upon request after publication through a collaborative process. Researchers should provide a methodically sound proposal with specific objectives in an approval proposal. Please contact the corresponding author for additional information.

## Supplementary Materials

The following supporting information can be downloaded at: https://www.mdpi.com/article/10.3390/jpm13071143/s1, Table S1: PRISMA checklist; Table S2: Search strategy.
