# Supplementary material for: Effects of Vasopressin Receptor Agonists during the Resuscitation of Hemorrhagic Shock: A Systematic Review and Meta-Analysis of Experimental and Clinical Studies
Source: J Pers Med. 2023 Jul 16;13(7):1143. doi: 10.3390/jpm13071143 (PMC10381354; doi:10.3390/jpm13071143)
Supplement: Supplementary file 1 [file jpm-13-01143-s001.zip › Table S2.pdf]

PUBMED

("shock, hemorrhagic"[MeSH Terms] OR "hemorrhage"[MeSH Terms] OR "shock"[MeSH Terms] OR "vasopressins"[MeSH Terms] OR "arginine vasopressin"[MeSH Terms] OR "terlipressin"[MeSH Terms] OR "lypressin"[MeSH Terms] OR "vasopressin analogue"[Title/Abstract] OR "argipressin"[Title/Abstract] OR "AVP"[Title/Abstract] OR "vasopressin receptor"[Title/Abstract] OR "V1 agonist"[Title/Abstract] OR "V2 agonist"[Title/Abstract] OR "lysine vasopressin"[Title/Abstract] OR "phenypressin"[Title/Abstract] OR "hemorr*"[Title/Abstract] OR "vasopr*"[Title/Abstract]) AND ("hemodynamics"[MeSH Terms] OR "cardiac output"[MeSH Terms] OR "stroke volume"[MeSH Terms] OR "vascular resistance"[MeSH Terms] OR "arterial pressure"[MeSH Terms] OR "blood circulation"[MeSH Terms] OR "microcirculation"[MeSH Terms] OR "cardiovascular dynamics"[Title/Abstract] OR "preload"[Title/Abstract] OR "venous return"[Title/Abstract] OR "heart-lung interactions"[Title/Abstract] OR "cardiac index"[Title/Abstract] OR "afterload"[Title/Abstract] OR "pulse pressure"[Title/Abstract] OR "blood flow"[Title/Abstract] OR "complications"[Title/Abstract] OR "outcome"[Title/Abstract] OR "mortality"[Title/Abstract] OR "survival"[Title/Abstract] OR "perfusion"[Title/Abstract])

- ➔ Pubmed: 220,072 results - PubMed Central: 6,859 results

SCOPUS

TITLE-ABS ("hemorrhagic shock" OR "hemorrhage" OR "hypovolemic shock" OR "vasopressins" OR "arginine vasopressin" OR "terlipressin" OR "lypressin" OR "vasopressin analogue" OR "argipressin" OR "AVP" OR "vasopressin receptor" OR "V1 agonist" OR "V2 agonist" OR "lysine vasopressin" OR "phenypressin" OR "hemorr*" OR "vasopr*") AND TITLE-ABS ("hemodynamics" OR "cardiac output" OR "stroke volume" OR "systemic vascular resistance" OR "arterial pressure" OR "blood circulation" OR "microcirculation" OR "cardiovascular dynamics" OR "preload" OR "venous return" OR "heart-lung interactions" OR "cardiac index" OR "afterload" OR "pulse pressure" OR "blood flow" OR "complications" OR "outcome" OR "mortality" OR "survival" OR "perfusion ")

- ➔ 166,915 results

CLINICAL TRIALS.GOV

vasopressin OR arginine vasopressin OR argipressin OR vasopressin receptor OR V1 agonist OR V2 agonist OR terlipressin OR lysine vasopressin OR lypressin OR phenypressin OR hemorrhage OR hemorrhagic shock

- ➔ 5,800 results
